# Endoscopic Detection and Management of Esophagogastric Varices

**DOI:** 10.7759/cureus.16825

**Published:** 2021-08-02

**Authors:** Ramila Shrestha, Jiwan Thapa, Bhuwneshwer Yadav, Bhawesh Thapa, Mukesh S Paudel

**Affiliations:** 1 Gastroenterology, National Academy of Medical Sciences, Kathmandu, NPL

**Keywords:** endoscopy, gastric varices, esophageal varices, band ligation, glue injection

## Abstract

Introduction

Gastrointestinal (GI) varices are abnormally dilated submucosal veins in the digestive tract caused due to portal hypertension. Esophagus and stomach are common locations of varices induced by portal hypertension. Their presence correlates with the severity of the liver disease. Endoscopic variceal band ligation is one of the preferred methods for bleeding and nonbleeding large varices to decrease bleeding risk. Tissue adhesives such as N-butyl-2-cyanoacrylate have been used for gastric variceal obturation.

Methods

This descriptive study was conducted in the Department of Gastroenterology, National Academy of Medical Sciences, Kathmandu, Nepal, from March 2014 to January 2020. The endoscopic detection of esophageal and gastric varices was observed. Endoscopic variceal ligation (EVL) was done for esophageal varices and injection of N-butyl 2-cyanoacrylate for gastric varices.

Results

Esopahageal varices were detected in 1266 patients (8%) and gastric varices were in 36 patients (0.2%) among 15,657 patients undergoing upper gastrointestinal (UGI) endoscopy. Nine hundred seven (71.6%) were male. Large esophageal varices were endoscopically detected in 54.8% patients, small varices in 31.4% and both (large and small varices) in 13.4%. EVL was done in 30.7% and EVL with cyanoacrylate glue injection in 35 patients (2.7%).

Conclusion

Esophageal and gastric varices are seen commonly in patients with chronic liver disease. This study was conducted to describe the different types of GI varices in patients undergoing UGI endoscopy. Variceal band ligation for esophageal varices and glue injection for gastric varices are viable options of management.

## Introduction

Gastroesophageal varices (GOV) are seen in approximately half of the patients with cirrhosis of the liver. As the severity of liver disease increases, the prevalence of varices also increases. Small varices progress to large varices at a rate of 10%-12% annually [1]. Gastric varices bleed less frequently than esophageal varices and are responsible for 10%-30% of all variceal haemorrhages. However, gastric variceal bleeding tends to be more severe with higher mortality [2]. Upper gastrointestinal (UGI) endoscopy is considered as the gold standard for the diagnosis of GOVs. Although esophageal varices are easy to detect, gastric varices often pose difficulty in identification [3]. Endoscopically, esophageal varices are classified into small (<5 mm) and large (>5 mm) [4,5].

The formation of gastric varices in association with portal hypertension was first described by Stadelmann in 1913 [6]. According to Sarin classification, gastric varices are categorized into four types based on their relationship with esophageal varices as well as by their location in the stomach: GOV type 1 (GOV1; extension of esophageal varices along lesser curvature), GOV type 2 (GOV2; extension of esophageal varices along greater curvature), isolated gastric varices (IGV) type 1 (varix in the fundus) and IGV type 2 (varices in stomach or duodenum) [2]. Endoscopic therapy is the only treatment modality that is widely accepted for the prevention of variceal bleeding, control of acute variceal bleeding and prevention of variceal rebleeding. Endoscopic variceal ligation (EVL) is simple to perform. Multi-band devices are used to deploy bands around the varices. EVL can cause local complications including esophageal ulcers and dysmotility [7]. The preferred endoscopic therapy for gastric variceal bleeding is an injection of polymers of cyanoacrylate usually N-butyl-2-cyanoacrylate [8].

In this study, we have reviewed the cases of esophageal and gastric varices that were detected and managed at our centre over a period of six years.

## Materials and methods

This descriptive study of retrospectively collected data was conducted at the Department of Gastroenterology, National Academy of Medical Sciences, Kathmandu, Nepal, from March 2014 to January 2020. All patients who presented to the endoscopy suite with various indications for endoscopy were screened for inclusion in the study. The study included patients above 18 years of age with endoscopically detected esophageal varices only or with gastric varices. After taking informed consent, UGI endoscopy was performed for the first time. The oropharynx was sprayed with 2% xylocaine and the patients were placed in the left lateral position. The mouth gag was then placed between the incisor teeth. The gastroscope (Fujinon 220) was then introduced under direct vision into the UGI tract. A total of 1260 patients were recruited.

Data were collected regarding the different sizes of esophageal varices in both sex and different age groups and the treatment modality for UGI bleeding. The study excluded cases of UGI bleeding due to isolated gastric varices; non-variceal causes like peptic ulcer disease, neoplasia, exposed eroded vessel, traumatic, iatrogenic, Mallory-Weiss tear, erosive gastritis; patients needing resuscitation; patients with acute GI bleeding that originated in the small intestine, colon and rectum and patients with obscured GI bleeding.

Esophageal varices were described as large (>5 mm) or small (<5 mm). Variceal band ligation was performed when indicated. Endoscopic variceal ligation is a procedure that involves suctioning of the varix into the cylinder of the banding device at the tip of endoscope and deploying a band around the varix. Multi-band devices are used to apply several bands over the oesophagal varices. Varices near the gastroesophageal junction were banded initially and then more proximal varices were banded in a spiral manner. For GOV2, esophageal variceal ligation was done only after glue obliteration of fundal varices.

Gastric varices were described as per Sarin's classification [2]. Glue injection was done for gastric varices when indicated. After puncturing the gastric varix with a needle, cyanoacrylate glue was injected in 1- to 1.5-ml aliquots by using normal saline or sterile water (about 0.8-1.0 ml) to flush the glue into the varix. As the needle was withdrawn from the varix, a steady stream of the flush solution was aimed at the puncture site. Additional glue was injected until the varix was hard to palpate.

## Results

A total of 15,657 patients underwent UGI endoscopy from March 2014 to January 2020 at our centre. Out of these patients, 1266 (8%) with a presence of varices were included in the study. The median age of patients was 45.5 years. All patients had esophageal varices. Both esophageal and gastric varices were seen in 36 patients. None of the patients in our study had isolated gastric varices. Among patients with varices, 907 (71.6%) were male. Among all varices, large, small and large with small varices were found in 54.4%, 31.4% and 13.1% patients, respectively (Table 1).

**Table 1:** Size of esophageal varices according to sex and age

Variable	Category	Number (%)	Size of esophageal varices		
			Large	Small	Both
			694 (54.8%)	398 (31.4%)	174 (13.4%)
Sex	Male	907 (71.6%)	497 (54.80%)	294 (32.41%)	116 (12.89%)
	Female	359 (28.4%)	197 (54.87%)	104 (28.97%)	58 (16.16%)
Age in years	<21	45 (3.5%)	29 (64.44%)	11 (24.45%)	5 (11.11%)
	21–30	100 (7.9%)	52 (52%)	25 (25%)	23 (23%)
	31–40	262 (20.7%)	140 (53.44%)	84 (32.06%)	38 (14.50%)
	41–50	389 (30.7%)	219 (56.30%)	123 (31.62%)	47 (12.08%)
	51–60	264 (20.8%)	146 (55.30%)	87 (32.95%)	31 (11.75%)
	61–70	139 (10.9%)	65 (46.76%)	53 (38.13%)	21 (15.11%)
	>70	67 (5.2%)	43 (64.18%)	15 (22.39%)	9 (13.43%)

Approximately 31% of patients had esophageal varices in the 41- to 50-year age group. There was a higher number of patients with large varices than small or mixed (large and small) varices in all age groups. Esophageal band ligation was done in 389 patients (30.7%) among all bleeding esophageal varices cases. Three to five bands were deployed over varices on an average. Once bleeding gastric varices were detected, cyanoacrylate glue was injected in 35 cases. EVL alone was done in 341 patients (91.1 %) whereas EVL with glue injection were performed in 8.82% of cases with large varices (Table 2).

**Table 2:** Endoscopic management in relation to the size of esophageal varices

Variable	Category	Endoscopic variceal ligation (n=389)	Endoscopic variceal ligation with cyanoacrylate glue injection (n=35)
Size	Large varices (n=374)	341 (91.18%)	33 (8.82%)
	Small varices (n=9)	7 (77.78%)	2 (22.22%)
	Large and small varices (n=41)	41 (100%)	0

In patients with small varices, 77.8% underwent EVL only, and combined EVL with glue injection was done in 22.2%. EVL was done in 41 patients (100%) with large and small varices.

## Discussion

We analysed the endoscopic findings in 1266 patients with esophageal and gastric varices who underwent upper gastrointestinal endoscopy between March 2014 to January 2020 at our centre. The endoscopic findings of esophageal varies were present in 8% of all endoscopies in our centre. In a similar study from Sudan, varices were present in 13.8% cases [9]. Among patients with cirrhosis and UGI bleedings, varices were diagnosed in 96.4% [10]. In another study conducted at our centre, variceal bleeding accounted for 23% of cases of acute UGI bleeding [11].

More than 90% of cirrhotic patients develop esophageal varices at some time in their lifetime and 30% of these bleed. Varices are present in about 30%-40% of compensated cirrhosis and 60% of those who present with ascites [12]. The progression from small to large varices occurs in 10%-20% of cases after one year [13]. In the two years following the first detection of esopahageal varices, the risk of variceal bleeding ranges from 20% to 30% [12-14]. In studies from Nepal, esophageal varices among UGI bleeding cases were found to be significantly higher in Mongolian origin cases due to increased consumption of alcohol [15,16]. In our study, large varices were observed in 694 patients (54.8%), small varices in 398 patients (31.4%) and both in 174 patients (13.4%).

In a study from Nepal, most patients of Child Turcotte Pugh (CTP) class B and C had large varices whereas those with CTP class A had small varices. Among 97 patients, 30 (30.9%) were in Child Turcotte Pugh class A, 30 (30.9%) in CTP class B and 37 (38.1 %) were in CTP class C. Small varices were found in 25 (8%) patients; 52% of patients had large varices with red color sign and 20.6% had large varices without red color sign [16]. In another study, the prevalence of esophageal varices was higher in CTP class C [17,18].

The first reported case of endoscopic variceal ligation performed on dogs in 1986 had a 92% success rate. After its safety and efficacy were assessed, it became available for use in humans in 1988 [19]. In a meta-analysis of eight randomized controlled trials involving 596 patients, EVL compared with beta-blockers reduced the rate of the first variceal bleed [20]. Variceal band ligation has also been shown to eradicate esophageal varices with fewer complications and lower rebleeding rates [21]. The incidence of gastric varices in patients with portal hypertension has been variably reported (2%-70%) probably due to difficulties in diagnosis [22].

Endoscopic glue injection is the first line of treatment in the management of acute gastric variceal bleeding, especially GOV2 and IGV1. However, glue injection comes with the risk of severe complications including venous and systemic thromboembolism (pulmonary embolism, stroke), ulcers with protracted bleeding and splenic and portal vein thrombosis [23]. Sarin et al. reported that primary gastric varices were seen in 114 (20%) patients among 568 patients (393 bleeders and 175 non-bleeders). Gastric varices (compared with esophageal varices) bled in significantly fewer patients (25% vs 64% respectively). Gastric varices had a lower bleeding risk factor than esophageal varices but bled more severely. Once a varix bled, mortality was more likely (45%) in gastric varix patients [2]. A study was also done to compare band ligation and cyanoacrylate glue injection in patients with bleeding due to gastric varices [24]. Better results were noted in terms of control, rebleeding and eradication of varices with cyanoacrylate glue injection. Similarly, Faheem et al. compared two groups (Group I, treatment with cyanoacrylate glue injection; Group II, treatment with endoscopic band ligation) with UGI bleeding due to gastric varices. Hemostasis was achieved in all patients (95%) of Group I [25]. Isolated gastric varices were not seen in our study.

## Conclusions

Endoscopy plays a major role in the diagnosis and management of gastrointestinal varices in portal hypertension. This study was conducted to describe the different types of gastrointestinal varices in patients undergoing UGI endoscopy. Esophageal varices were the most common type of varices at our centre.

Esophageal and gastric varices are seen commonly in patients with chronic liver disease. Variceal band ligation for esophageal varices and glue injection for gastric varices are viable options of management.
